# The Animal Suture; Its Use in Surgery

**Published:** 1890-04

**Authors:** Henry O. Marcy

**Affiliations:** Boston


### THE ANIMAL SUTURE; ITS PLACE IN SURGERY.

By Henry O. Marcy, M.D.,

Boston.

Investigations upon the origin of means adapted to a given end clearly teach that active minds have already subjected the chief factors to careful analytical study, although certain phases of the problem are ever presenting themselves under new aspects. Dominated by such thought, Solomon taught that "there was no new thing under the sun," and it is quite probable that the animal ligature was known and used by the Egyptian surgeons at a period antedating this wise Jewish philosopher.

The connective tissue of animals was early utilized for a great variety of purposes when it was necessary to secure great strength and high tension. The Homeric poems afford familiar illustration. In the Odyssey, the strings of the old Greek harp are described as made from the twisted intestine of the sheep. The ancient Egyptian harp is said to have been strung in a similar manner. The celebrated Arabic writer, Rhazes, who practised in Bagdad, A.D. 900, describes the stitching of wounds of the abdomen with the strings of the harp, and Albucasis also mentions the stitching together of wounds of the bowel with a fine thread from the twisted intestine of an animal.

The careful student of the early history of surgery finds abundant evidence that the ligature was used as a hemostatic agent at a very early date. Celsus, at the end of the first century, describes the ligature as of ancient origin, and says that it was used by the Alexandrian school of medicine, with the teachings of which he seems to have been familiar. He advised placing two ligatures upon the vessel and dividing it between the points of tying. Galen recommended its use, and Vesalius, in the sixteenth century, mentions the ligature as a relic of the past, greatly underestimated in value because of the lack of anatomical knowledge. Its first application in amputations, on account of gunshot wounds, was doubtless by Ambrose Paré. At this time, wounds were cauterized with boiling oil, in the belief that gunpowder in some way poisoned the wound.

Special studies, however, for demonstrating the value of the material used for ligatures do not appear to have been made in he early history of surgery. Indeed, there is little doubt that, whatever the material used, it was always considered as a foreign body to be ultimately eliminated from the wound, and the material, selected for its strength without special reference to the irritation which might be induced thereby, naturally commended itself in the form of thread, silk, or hemp, which was in ordinary domestic use.

To our distinguished countryman, Professor Physick, of the University of Pennsylvania, is undoubtedly due the honor of having first introduced what is known as the *animal ligature* into surgical practice. His ligatures were made of chamois leather, and he and the late Dr. Dorsey usually rolled their ligatures on a marble slab to make them hard and round. The advantages claimed for the ligatures by Dr. Physick were that, being made of animal matter, they would serve long enough to obliterate the artery and be speedily removed by the absorbents, thus avoiding the difficulty arising from a foreign body, however minute. These ligatures were used in this country to a great extent, and Sir Astley Cooper demonstrated their superiority in his own operations.

"EXPERIMENT I.—Twenty-two days after tying the carotid artery of a sheep with buckskin ligature the wound was found entirely healed, the artery obliterated, upon one side of which was a little yellowish matter which proved to be a portion of the ligature.

"EXPERIMENT 2.—Twenty-five days after tying the left carotid of a dog, there was seen on either side of the vessel two little yellowish knobs, which proved to be the two ends of the ligature in the state of yellow pulp. The knobs were completely enveloped in a strong membranous capsule, the same capsule enclosing both knobs, and its fibres plainly seen crossing over the artery. * * * On the lower side, where the coats were not cut, in splitting open the vessel to examine its interior, there was an actual adhesion of the leather to the inner coat. The string was softened into a pulpy consistence, and near the inside of the vessel was evidently covered with organized lymph, and extended along its edge on one side some distance. When the string was gently pulled, one plainly saw a delicate membrane, passing off from the artery and ending on the string in fringed extremities. This attachment of the vessel by a new formation existed on both sides of the artery. The capsule covering the knobs or ends of the string was fully equal in strength to the outer coat of the artery, and therefore there was no tendency to hemorrhage.[1]"

In all the essentials the experiments of Dr. Jamieson were not unlike those of Sir Joseph Lister, repeated half a century later; and the former at a period when so little was expected of the American people in the way of literary productions, to say nothing of scientific research, that one of England's famous critics asked, "Who reads an American book?"

Dr. Ephraim McDowell, in 1809, tied the pedicle of his first case of ovariotomy with a ligature of buckskin.[2]

In the history of ovariotomy in the United States, by the late Dr. Peaslee, it is stated that Dr. Nathan Smith, Professor of Surgery in Yale College in 1821, tied the arteries with leather ligatures (narrow strips cut from a kid glove), which were returned into the peritoneal cavity and the incision was closed, followed by recovery.

[1] Cooper's Medical Dictionary, sixth American edition.

[2] It is probable that Dr. McDowell, and other operators later, made use of fine strings cut from the skin of the deer, which had been tanned after the methods of the Indians, then and for a long time thereafter, in common use. These gave a ligature greatly superior to that cut from skins tanned by the modern methods.

Professor Paul F. Eve, of Nashville, Tennessee, wrote in 1876: "I have been in the habit of using the sinews of the deer, for ligating vessels, for forty years. I have never used carbolized spray. The tendons of the deer dried and torn into shreds and rolled into ligatures are what I employ. They are absorbed; I have occasionally used them as sutures, but generally apply silk for this purpose."

These fragmentary experiences, drifting down to us through the years, teach that there was more or less blind groping after a something that should serve a better purpose than that which the routine of daily practice, in the use of the hemp or silken ligature, afforded. It was reserved for the present generation to make possible a scientific basis for the better consideration of ligatures and sutures in their application to the living structures. In the light of our present knowledge of surgical pathology, the opposition to the ligature in the days of Ambrose Paré, which we have been wont to attribute to the conservatism of ignorance and stupidity, is invested with a new and vital interest. The amputated limb, seared with the hot iron, as a hemostatic, a measure most barbarous and revolting, gave as a result an aseptic wound. Repair was necessarily slow and tedious, but abundant granulation supervened before decomposition ensued, to protect from septic absorption.

The constricting ligature, the septic, pocketed wound, with little care as to cleanliness, gave such secondary fatal results that we are led to wonder that the innovation of the ligature, in the closing of the great vessels, became the established practice. Had it not been for the frightful dangers from secondary hemorrhage after the use of the cautery, slow healing giving imperfect results, it may well be questioned if even the indomitable spirit of Ambrose Paré could have made the innovation survive his own time.

A deeper philosophy sought solution of the problem as to the causation of suppuration in wounds, and if its prevention was not within the possibility of the rule, rather than the exception. The studies of Pasteur, Tyndall, our own Jeffries Wyman and others, undertaken for the solution of the problem of spontaneous generation, brought fruitage to the human race little dreamed of by these wise philosophers.

The genius of Mr. Lister seized upon the application of the thought, and with a patient, investigating spirit and painstaking toil, worked out the fundamental factors of the *rôle* of ferments in wounds. It was not until rules could be formulated, based upon the scientific deduction that operative wounds should be free from suppurative processes, hitherto considered almost necessary concomitants, that the proper conditions for the study of ligatures and sutures were rendered possible. Of necessity in intimate association with the question of the treatment of operative wounds, arose, *de novo*, a most interesting chapter devoted to the best means of controlling arterial hemorrhage.

It was clear that the hitherto prevailing method of ligation, leaving the ends of the ligature long, extending from the wound, by so much at least prevented primary union; while cutting the ligature short, and closure of the wound, were fraught ever with disastrous consequences, when the septic ferments were thereby deeply buried. When aseptically applied, the constricting silken ligature too often proved an irritating foreign body, to be ultimately slowly eliminated.

In retrospect, with present knowledge, what seems simple factorage of the problem proved extremely difficult of solution. The conservatism of thought, the prejudices of the large number of the surgical authorities of the time, wedded to present measures, misled by other phases of dominating thought—the so-called vital processes of inflammation, irritation, cell proliferation, etc.—engrossed the subject with many difficulties. The demonstration that fermentation and suppuration in a wound resulted from the introduction of a *something* from without, was the first real step of progress. To eliminate that something was the next problem for solution. It was clearly shown that the torsion of an artery to procure rupture and intrafolding of its interior coat, causes permanent closure of the vessel, and that the living structures, unpoisoned by germ infection, possess the power of easy disposition of the aseptic necrotic portion devitalized by violence.

Histological study demonstrated that the necrosed part no longer underwent the changes which had formerly been supposed necessary for the elimination of dead material, known as suppuration, gangrene, etc., but that the part becomes invaded by living cells which, little by little, produce a local change marked by early disappearance of the necrosed tissue. This naturally led up to the thought if extraneous animal tissue could not be prepared in a way that, introduced into the vitalized structures, would be followed by a similar result.

Repeated experimentation taught that small pieces of dead tissue, preserved in carbolic acid solutions, incorporated into the living structures, were disposed of in a manner not unlike the necrosed portion of a twisted artery, and led to the inference that animal tissues properly preserved could be used for the constriction of vessels. In looking about for suitable material of animal type to be used as ligature, the catgut prepared for musical purposes naturally offered itself. It proved comparatively easy, by immersion for a considerable period in a carbolic solution, to render the material non-infective, but, as a soft, slippery strand, it lacked necessary qualities in which to make a secure knot, and by its early softening in the tissues, loosened and thereby failed to secure the end sought. A long immersion in an oily solution of carbolic acid, to which a very little water had been added, produced a material change of structure—a kind of tanning process thereby resulted, which gave to the material the quality of less easy softening in the tissues, as well as retaining a firm knot, which was ultimately disposed of by the surrounding structures.

I know of no series of experimental studies better worthy of admiration, or emulation, than Mr. Lister's experiments undertaken for this purpose upon the lower animals, which the laws of England necessitated his resorting to the Continent in order to make possible. The resulting changes, histologically shown to have taken place in ligatures applied to the vessels of the lower animals examined at different periods, taught that the cell proliferation at first enclosed, and later on penetrated, the carbolized ligature, until, little by little, it became replaced by living connective-tissue cells—a discovery the importance of which is scarcely fully appreciated, even to the present time. Mr. Lister's experiments were limited to the ligation of vessels, and there has resulted from his teachings the surgical treatment of the great arterial system with a safety hitherto deemed impossible. The larger vessels are now tied in continuity, in close relation to their bifurcations, even the greater trunks, with a seeming impunity little less than startling.

Returning in 1870 from my studies at Edinburgh under Mr. Lister, liberally supplied with a variety of the materials which he then advised in operative treatment, I not only made use of catgut for the ligation of vessels, but accident early furnished me the opportunity for a new application of the ligature, in the form of buried sutures. February 19, 1871, I closed the ring, necessarily greatly enlarged for the reduction of a strangulated hernia, with deep sutures of catgut. This I did in order to retain the abdominal contents, because of a severe asthmatic bronchitis from which the patient was also a sufferer. The resultant permanent cure of the hernia, with a marked proliferation of tissue along the line of the buried sutures, led me to inquire if the sutures buried in the part had not been disposed of in a manner similar to that demonstrated by Mr. Lister, resulting about the catgut ligature surrounding the arteries.

I immediately instituted a series of experimental histological studies upon the lower animals, and demonstrated that, along the track of an aseptically buried suture, cell proliferation rapidly supervenes and that new cells invade the softened tissue, and *pari passu* with its absorption, a *living band of connective-tissue cells* replaces the whole line of the suture. If rapidly absorbed, the proliferated cells are minimized ; as the process goes on more slowly the change becomes more distinctive until, in young animals, in ten to fifteen days all trace of the suture as such is lost. The value of such reinforcement of the tissue along the line of the sutures became at once apparent in their application to the cure of hernia, and, little by little, I early extended their use to the closure of wounds of every description, publishing, from time to time, my results.

In the pursuance of my studies, I early had occasion to examine a great variety of the specimens of catgut offered in the market, although from the first I adopted what seemed to me the wise precaution of preparing my own sutures. In catgut there are, of necessity, certain inherent defects. Its method of preparation is not generally known to the profession, who have rarely questioned the product beyond the condition in which it is offered for sale prepared for the musician. The best of these varieties usually come from Italy, prepared from the intestine of the sheep of the mountainous districts. The small intestine necessarily undergoes maceration until the connective-tissue layer, which, as a loose fibrous sheath, unites the mucous and muscular coats of the intestine, is softened, and can be easily separated in a manner not unlike that practised in the preparation of the intestine of the pig for the making of sausages. This is split by a cork, armed with sharp blades, drawn through the circular sheath, dividing it into sections to produce the desired size. These ribbons are twisted, dried, and oftentimes sandpapered to give evenness to surface, and usually put up in little skeins from twelve to fifteen feet in length—the catgut of commerce.

The connective tissue cells of the fibrous coat of the intestine are irregularly disposed, the fine fibrils more commonly crossing diagonally to the longitudinal axis of the intestine, a wise distribution of this strengthening portion of the intestine to allow considerable change in its shape. When carefully examined, under a low-power lens, the fibres are seen to be irregularly interlaced, not unlike a strip of cloth cut diagonally. The gut, even in the dry state, has a perceptible yield on tension, and every musician knows the care requisite to protect his strings against moisture. Frequent allusion is made in the classics to the care demanded of the bowman in this respect, when it was customary to string the weapon with animal products.

Macerate a piece of catgut until it can be easily unfolded, the above condition is readily apparent. Moreover, its division is rarely uniform, and, when sandpapered, the removal of the irregular projections causes oftentimes large abrasions, or rents. No matter how prepared for surgical use, ultimately the result obtained will depend, in considerable measure, upon the integrity of its structure, since the component cells are, little by little, separated by the penetration of the new proliferating tissue. The long maceration in the first stage of preparation, remaining for a considerable time a putrefying mass, necessarily damages the material, not only by softening the adhesion of the cells, but infecting them with bacteria; and in the use of the catgut for all surgical purposes it is important, as the first step in preparation, to destroy any germ infection that may remain. After this has been effected, no method which I have tried gives a result equal to that formulated by Sir Joseph Lister.

It improves by age and is better not to be used until after it has been several months in carbolic oil. The preliminary disinfection of the gut is of the first importance, since preservation of the hardened structure in the carbolized oil may not penetrate to the destruction of bacteria within the strands.

I have elsewhere published in detail the micrococcal infection developing only along the line of the buried sutures, of four consecutive surgical cases, giving evidence upon which I deduce the conclusion that it could have been only to this inherent defect of the catgut, which had been selected from freshly opened preparations, preserved in carbolic oil, which were sent me from London.

Dr. Macewen published a very interesting article giving his method of preparing catgut, so as to render it more reliable, by immersing it in a watery solution of chromic acid—1 to 5—and adding one part of this to twenty of glycerine. Remove in two months and preserve ready for use in carbolic acid and glycerine—1 to 5. By varying the strength, gut is prepared possessing different degrees of resistance to absorption in the tissues. My own experience of animal sutures prepared in this way has not been satisfactory.

I have also used catgut prepared in oil of juniper and kept in alcohol, but think it inferior to chromicized gut.

Owing to these inherent defects in catgut, in common with other surgeons I was led to inquire if there were not other animal tissues better suited for surgical purposes.

The tendinous structures of the body present the connective tissue cells parallel and firmly united to each other. Although generally thus disposed, there is considerable variety in the arrangement of the cells, making a parallel separation much more uniform in some tendons than in others. As far as possible, a detailed investigation of animal tendons of sufficient size has been prosecuted with varying success.

The tendons of the hind leg of the moose and caribou, soaked in a sublimate solution until soft, were the first tested. A considerable portion of the tendon may be subdivided sufficiently fine for sutures, in length from fifteen to eighteen inches.

Dr. John H. Gilman, of Lowell, called my attention to tendons from the whale, stating he "had used them with great satisfaction in the ligation of vessels." Specimens were sent me from Provincetown four feet in length and of sufficient strength to draw a cart, but the ultimate fibrils were interlacing, while the whole tendon was interspersed with adipose cells. I obtained ligatures also from the whale tendon which were made under the direction of Dr. T. Ishigure, of Tokio, Surgeon-in-Chief of the Imperial Japanese Army. The mode of preparation is given as follows: "First, a whale's tendon is dissected by the points of needles, and teased out until the fibres look very like those of hemp. Secondly, the longest and finest fibres among them are selected, and they are then spun together as ordinary silk thread." There can be no question ligatures thus prepared are very serviceable, but the specimens furnished me were not suitable for sutures.

The Sioux Indian women in the Northwest taught me, in 1882, their manner of sewing buffalo skins with the tendinous structures derived from the fascia lata of the buffalo, which they preserve for this purpose by drying and smoking. During the present Summer, I obtained from Mr. Harry Adams, of the Hudson Bay Company, in Winnipeg, Manitoba, specimens from the fascia lata of the moose prepared by the Indians of the far Northwest Territories, as a substitute for that from the buffalo now extinct, called by them *Astis*. They use it in the dry state, stripping it as they sew, occasionally wetting it in the mouth. Good tendon sutures in any quantity can be obtained from this source. My specimens, however, are not more than fifteen inches long, and are quite inferior to the kangaroo tendon. Some years since, a distinguished Russian surgeon sent me similar specimens from the reindeer, finely divided and slightly twisted. These I prepared and used with satisfaction.

In 1880, Dr. S. G. Simmons, of Charleston, S. C., sent me admirable specimens of tendons from the tail of the fox squirrel, with the statement that he had often used them for delicate surgical purposes with great satisfaction. This tendon is composed of exquisitely beautiful parallel fibrils, which can be subdivided without fraying to the size of fine threads. Their extreme length, however, scarcely exceeds nine inches. Reasoning from comparative studies, it seemed to me highly probable that the kangaroo should possess a tendon, similar in quality, traversing the entire length of the tail. Through the kindness of the late Mr. Alonzo H. Newell, of Boston, for many years a prominent merchant in Australia, I obtained some most excellent specimens. At the International Medical Congress held in London, in 1881, in a paper upon the cure of hernia I mentioned the use of the tendon suture from the kangaroo and other animals as especially to be commended. Reference to my recommendation of the kangaroo tendon and its value in surgery was some time later made in an Australian publication. This came to the notice of Dr. Girdlestone, who wrote me that he had used kangaroo tendons for some years with great satisfaction, and had published the results.[1]

The tendons should be taken from recently killed animals, quickly sun-dried, and kept dry until ready for further preparation. This prevents primary decomposition, which we have pointed out as necessary in the preparation of the catgut. When soaked until soft they are easily separated as fine as desired, with remarkably little waste, and give threads from the size of hairs upward, and from eighteen inches to two feet in length, exceptional specimens being even considerably longer. The kangaroos are so numerous as to make their skins of commercial value, being sent to the United States in lots each of many thousands, yet it is only with the greatest difficulty and expense that I have succeeded in securing tendons sufficient for my own use, although I have sent *carte blanche* orders to various parties in Australia. I was informed they could be obtained in London, but two years ago the enterprising firm of Grosvenor & Richards, of Boston, searched the London markets without avail. Quite recently, Felton, Grimwalde & Co., of Melbourne, Australia, wrote me that they regularly keep in stock tendons prepared for surgical purposes. I cannot doubt that, at an early date, surgeons will be furnished with the kangaroo tendons, which I fully believe are in every way greatly to be preferred to catgut.

Dr. Dudley,[2] of Texas, has written a most interesting article upon the use of the tendon of the lepus, or mule-eared rabbit, as a material for ligatures and sutures. Dr. Dudley does not state the portion of the animal from which he obtains the tendon, but describes them as, "an aponeurosis of muscles rolled upon each other, susceptible of being torn into minute threads, if so desired." He first had occasion to use the tendon of the lepus as a suture, in the fresh state, in 1881, finding he had no silk in his pocket-case. He has continued the use of these tendons with the greatest satisfaction to the time of his report.

The use of animal *suture* requires the same and the only precautions that are requisite to the successful application of the ligature. It *must be* in *itself aseptic*; it must be *aseptically applied* in an *aseptic wound*. When thus applied, the range of its uses may

[1] Tendon Ligatures. T. M. Girdlestone. Australian Medical Journal, Melbourne, 1887, vol. xxii, page 356.

[2] Animal Ligatures and Sutures. H. W. Dudley, M.D., Hillsboro, Texas. Transactions Texas State Medical Association, 1884, page 133.

be extended to all operative wounds. It is difficult to conceive if any possible advantage is to be derived in the treatment of any aseptic wound by leaving it open—the so-called open-wound method.

Before the *rôle* of bacteria in wounds was understood, when it befell from chance, rather than scientific care, that primary union supervened, it is easy to understand how many who dreaded the daily experiences of fermentative material retained in pocketed wounds, not only refused to rely upon drainage with occasional irrigation, as a sufficient preventive of septic poisoning, but they insisted, as far as possible, upon allowing no recess in which purulent material could gather. In order to effect this, the lips of the wound were separated and kept apart by antiseptic dressings, so that the wound might heal by granulation from its very base. This is manifestly safer for the patient, and the result attained is not unlike that from the repair processes which supervene in the secondary healing of infected wounds; but those who advocate this method thereby confessedly acknowledge their lack of confidence in the modern methods of wound treatment, and their inability to protect wounds from infection.

In rare instances, it has been claimed that the resulting cicatricial union gives an increased strength to the parts involved, an opinion which it seems easy to show is unscientific and contrary to the general consensus of surgical opinion.

If it is correct to assume that the theoretic perfection in wound-treatment, which it is the ambition of the surgeon to attain, means a reunion of the divided parts, the anatomical relationship to be restored and maintained, then the suture holds a higher place in surgery than ever hitherto considered.

If the suture itself is replaced by vitalized structures, then its proper application becomes of the highest importance, the value of which the profession is only recently seeming to begin to realize. Given, as illustration, the joining of a divided retracted nerve or muscle, and its restoration to subsequent perfect usefulness; the sundered cervical tissues after a hysterectomy where the delicate joining of the peritoneum allows no open wound for hemorrhage or absorption; the reunion of the abdominal wound after laparotomy, where the peritoneum is independently united by a layer of buried sutures, and where the linea alba, or the muscular aponeurosis of the sheath of the recti is carefully rejoined, since the adoption of which method I have not had a single case of ventral hernia ; or, again, in the amputation of large tumors, where the remaining tissues are carefully coapted, so that retention and pocketing of fluid are impossible, rendering drainage not only superfluous but harmful.

I would not underestimate the importance of drainage in wounds that are necessarily septic, and in this class of wounds there is no advantage to be gained in the attempted use of the buried suture.

My own experience with the buried animal suture commenced with its use in a case of hernia in 1871, and this, with other cases where the cure was believed to be referable to the buried suture, was first published in the *Boston Medical and Surgical Journal*, November, 1871. In 1878 I contributed a paper on the subject at the meeting of the American Medical Association. In 1881 a further contribution upon the same subject, emphasizing the value of the tendon suture, was published in the *Transactions of the International Medical Congress.*

These and various other articles giving the results and surgical advantages of the use of the buried animal suture and its adaptability to special purposes, were reprinted and widely distributed to the profession, both in Europe and America.[1]

If the premises, which I have assumed in the early discussion of this paper, are correct--that a properly prepared aseptic animal suture aseptically applied, retains its strength sufficiently long to hold at rest the coapted parts until primary union is effected, and then itself slowly disappears, after having fulfilled this function, to be in a measure replaced by vitalized connective tissue—there can be little wanting to attain the theoretic perfection in the suturing of wounds. The first observations which I published, perhaps, naturally provoked only incredulity, and the results were

[1] Animal Ligatures. Annals of Anatomy and Surgery, July, 1881, page 232.

Cure of Hernia by the Antiseptic Use of Animal Ligatures. Transactions of International Medical Congress, 1881.

Animal Ligatures. New England Medical Monthly, June, 1883.

The Restoration of the Perineum by a New Method. Journal of the American Medical Association, Oct. 27th, 1883. Reprint.

The Surgical Advantages of the Buried Animal Suture. Journal of the American Medical Association. July 2d, 1885. Reprint.

The Perineum ; its Anatomy, Physiology, and Methods of Repair after Injury. Philadelphia : William J. Dornan, 1889.

A Treatise on Hernia. The Radical Cure by the Use of the Buried Antiseptic Animal Suture. Published by George S. Davis, Detroit, Mich., 1889.

considered at best accidental. The evidence already accumulated and presented to the profession by a great variety of observers in different parts of the civilized world is now sufficient to substantiate this claim.

The mode of application of the buried suture is of the greatest importance. Without exception it should be a continuous suture, since thus used more perfect coaptation of the parts can be obtained, while, as far as possible, knots should be avoided. It is important to minimize the material consistent with careful coaptation. It should join like tissues, periosteum to periosteum, muscle to muscle, deep fascia to deep fascia, and skin to skin, after deep incisions of all kinds. It should support the parts always without any constriction of tissue. Having secured these ends, it matters comparatively little by what manner of stitch it is applied. The opinions of operators will be modified in preference for the methods to which they have become accustomed.

For certain purposes it has seemed to me a manifest gain to include the tissues by a double suture, taken by means of a needle with eye near the point, which allows the ends of the suture to be introduced through the same puncture from opposite directions. This of necessity encloses and should coapt, but *must not constrict*, the tissues involved.

For wounds in the intestine and in resection, I have repeatedly used a double layer of tendon sutures, the Lembert stitch, taken as a continuous, not an interrupted, suture. The finer curved Hagedorn needles are the best. The needle pierces the peritoneum as in the Lembert stitch, entering and emerging about two lines from the sides of the wound. This intrafolds and coapts the peritoneum evenly. In resection of the intestine, or if an incised wound is long, I take a second layer about one-fourth of an inch outside of the first, coapting more peritoneum and thereby burying entirely the first line of sutures. Post-mortem intestine thus joined will hold water, and in a well-vitalized subject the effused lymph in a few hours entirely covers in the affected part. The advantages of this method are simplicity, rapidity of operation, avoidance of knots, a minimum of manipulation and of injury of the tissues involved, even and sure closure of the parts—all of which are of the first importance to a good result, as well as to the security and non-irritability of the tendon suture.

As far as possible, the suture should cross the wound at right or acute angles and not lie parallel with it. For the coaptation of large wounds, I have found it a decided advantage to use a running stitch, taken from side to side, both ends left externally free, tension upon which evenly coapts the sides of the wound. Fine tendons are thus introduced in two, three or more layers, commencing at the very base of the wound.

The skin is evenly coapted by a blind running stitch taken from within outward through the deeper layer only. This evenly unites the edges of the skin, while the suture itself is buried beneath it. The wound is dried, dusted with iodoform, covered with a layer of iodoform collodion, reinforced by a few fibres of absorbent cotton. The ends of the deep lines of the running sutures, after sufficient tension has been exercised to produce coaptation, are fixed in the collodion and cut off.

The subsequent dressing matters little ; a soft pad of cotton may give comfort by its support, but if the wound is *aseptic* at the close of the application of the iodoform collodion, it being presupposed that each step has been taken with modern aseptic precautions, *primary union will supervene, and drainage is not only needless, but is a positive disadvantage and danger.*

Aseptic wounds thus treated give really a simplicity of detail which seems surprising when contrasted with clumsy and bungling antiseptic dressings which this method—the aseptic application of buried animal sutures—is destined to supersede. In conclusion, the *rôle* of the buried animal suture may be accepted as a corollary to antiseptic surgery, upon the basic principles of which it is founded as a scientific deduction.

